
JOURNAL INFORMATION
==============================
NLM Title Abbreviation: Innov Surg Sci
Journal ID: Innov Surg Sci
NLM Journal ID: 101708165
Journal ID: iss

Innovative Surgical Sciences

EISSN: 2364-7485
Publisher: De Gruyter

ARTICLE INFORMATION
==============================
PMCID: PMC13068493
DOI: 10.1515/iss-2026-2009
Article ID: iss-2026-2009
Article version: 1
Subjects: Article

9. Spinal Surgery

Electronic publication date: 2026 Apr 10
Publication date: 2026 Apr
Volume: 11
Issue: Suppl1
First page: 428
Last page: 437
Copyright: © 2026 by Walter de Gruyter Berlin/Boston
Copyright year: 2026
Copyright holder: De Gruyter
License: This work is licensed under the Creative Commons Attribution 4.0 International License.
License URL: https://creativecommons.org/licenses/by/4.0/

Page count: 10

==============================

DE GRUYTER BRILL

**AO Spine Primary Spine Infection Validation by the AO Spine Knowledge Forum Trauma & Infection**
ID: E-DCK-390

**Wirbelsäulenchirurgie / Spinal Surgery**
**Infektionen der Wirbelsäule / Spinal Infections**

Klaus Schnake[1], Alexander Vaccaro[2], Jonathan Dalton[2], Rick Bransford[3], Charlotte Dandurand[4], Grace Xiong[5], Brian Karamian[6], Greg Schroeder[2]

[1]Zentrum für Wirbelsäulen- und Skoliosetherapie, Malteser Waldkrankenhaus St. Marien, Erlangen, Deutschland, [2]Department of Orthopaedic Surgery, Rothman Orthopaedic Institute at Thomas Jefferson University Hospital, Philadelphia, Vereinigte Staaten, [3]Department of Orthopaedics and Sports Medicine, Harborview Medical Center, University of Washington,, Seattle, Vereinigte Staaten, [4]Combined Neurosurgical and Orthopedic Spine Program, Vancouver General Hospital, University of British Columbia, Vancouver, Kanada, [5]Department of Orthopaedic Surgery, Stanford University, Redwood City, Vereinigte Staaten, [6]University of Utah, Department of Orthopaedic Surgery, Salt Lake City, Vereinigte Staaten

**Background:**
Primary spine infections (PSIs) are an increasing global health concern with significant morbidity/mortality—unfortunately, current classification systems are limited in scope. Consequently, the AO Spine Knowledge Forum Trauma & Infection (KF T&I) developed a comprehensive PSI classification system via a modified Delphi method. This system includes subcategories for infection location and morphology, neurologic status, and various modifiers. The purpose of this study was to perform a validation of this new system to assess its inter-rater and intra-rater reliability/accuracy among a group of international spine surgeon experts.

**Material and methods:**
Spine surgeons from the KF T&I independently classified 19 distinct clinical cases of PSIs using the new classification. Assessment was conducted at two separate time points to evaluate intra-rater reproducibility. A gold-standard classification for each case was established by a consensus of five KF T&I experts to serve as a benchmark for accuracy. Inter-rater reliability and intra-rater reproducibility were assessed using a modified Fleiss' Kappa statistic. Accuracy was calculated as the percentage of agreement with the gold standard.

**Results:**
After exclusions, 15 reviewers completed assessment 1 and assessment 2 of the 19 patient cases, for a total of 570 assessments. Fleiss' kappa was 0.76 in both assessment rounds regarding all locations/morphologies, indicating substantial interrater agreement. Agreement for the primary spinal region (A, B, C) was almost perfect ($\kappa = 0.98-0.99$). The average intra-rater reproducibility for the full classification was almost perfect (mean $\kappa = 0.90$), with 78.6% of raters demonstrating "almost perfect" reproducibility. The classification type with the lowest interrater reliability was C3 (mean $\kappa = 0.55$). The overall mean agreement with the gold standard for all locations/morphologies was high, at 87.4% in the first assessment and 87.7% in the second.

Open Access. © 2026 The Author(s), published by De Gruyter. This work is licensed under the Creative Commons Attribution 4.0 International License.

**Conclusion:**
The AO Spine Primary Spine Infection classification system demonstrated substantial inter-rater reliability and almost perfect intra-rater reproducibility in this validation study. These findings suggest it is a reliable and accurate tool for classifying PSIs. Future prospective studies are warranted to externally validate reproducibility and to assess its clinical utility in standardizing patient care globally.

DE GRUYTER BRILL

**MIS extreme lateral versus open oblique approach for lumbar vertebral body replacement**
ID: E-DCK-496

**Wirbelsäulenchirurgie / Spinal Surgery**
**Minimalinvasive Techniken / Minimally Invasive Techniques**

Maximilian Wankner[1], Krishnan Sircar[2], Nikolaus Kernich[2], Roland Goldbrunner[1], Volker Neuschmelting[1], Sergej Telentschak[1]

[1]Uniklinik Köln, Allgemeine Neurochirurgie, Köln, Deutschland, [2]Uniklinik Köln, Orthopädie und Unfallchirurgie, Köln, Deutschland

**Background:**
Direct comparisons between the MIS extreme lateral and standard open oblique lateral approaches to the lumbar spine in the context of corpectomy and vertebral body replacement remain scarce. This study aims to fill that gap by evaluating these two surgical techniques to inform clinical decision-making and optimize patient outcomes.

**Material and methods:**
The objective of this study was to compare clinical and surgical outcomes between the open oblique lateral and MIS extreme lateral retroperitoneal approaches to the lumbar spine for corpectomy and vertebral body replacement. The oblique lateral approach involved a wide exposure with prepsoas access to the vertebral body, while the extreme lateral approach utilized a smaller incision (mini-lumbotomy) with tubular retractors and a transpsoas corridor, guided by continuous EMG monitoring. This monocentric, retrospective study included 62 consecutive patients who underwent lumbar vertebral body replacement between 01/2017 and 03/2025 for indications such as osteoporosis, traumatic fracture, or spondylodiscitis, all via a lateral retroperitoneal approach. Using propensity score matching based on age, ASA score, BMI, primary vertebral pathology, and levels of vertebral body replacement, 22 patients were matched to each surgical group. Comparative analyses were performed using Fisher's exact test for categorical variables and the Wilcoxon signed-rank test for continuous variables, with statistical significance set at $p < 0.05$.

**Results:**
No significant differences were observed between the groups in terms of intra- or postoperative complications, new neurological deficit, blood loss, revision surgery rates, pain and opioid usage at discharge or mortality (all $p > 0.05$). However, operation duration was significantly longer for the extreme lateral approach, with a median of 145 minutes (IQR: 112–214), compared to 105 minutes (IQR: 87–132) for the oblique approach ($p = 0.002$).

**Conclusion:**
Both the oblique lateral and extreme lateral approaches to lumbar vertebral body replacement showed similar safety and clinical outcomes. The oblique approach had a significantly shorter operative time, suggesting greater efficiency. However, the extreme lateral approach may offer better cosmetic results due to a smaller incision and does not require a surgical assistant to hold retractors, which can simplify the procedure in certain settings.

DE GRUYTER BRILL

**Minimally Invasive Surgery: Outcomes in Intradural Extramedullary Spinal Tumors**
ID: E-DCK-97

**Wirbelsäulenchirurgie / Spinal Surgery**
**Minimalinvasive Techniken / Minimally Invasive Techniques**

Endrit Vinca[1], Dounia Hmimidi[1], Dieter Henrik Heiland[1], Daniel Delev[1], Oliver Schnell[1], Waseem Masalha[1]

[1]Neurochirurgische Klinik, Uniklinikum Erlangen, Deutschland

**Background:**
Surgical resection remains the gold standard for intradural extramedullary spinal tumors. Conventional open approaches often involve extensive muscle dissection and resection of posterior elements, which may lead to postoperative instability, delayed recovery, and higher complication rates. Minimally invasive spine surgery (MISS) offers a tissue-sparing alternative by preserving musculature and stabilizing structures, which is particularly relevant in tumor patients with otherwise intact spines.

**Material and methods:**
We performed a retrospective single-center analysis of 30 consecutive patients who underwent MISS for resection of intradural extramedullary spinal tumors over a two-year period. All procedures were carried out using tubular retractors and microscopic assistance. Clinical and imaging data were reviewed to assess neurological outcome, extent of resection, and perioperative complications. Outcomes were systematically compared to complication and resection rates reported for conventional open approaches.

**Results:**
Gross total resection (GTR) was achieved in 29 of 30 patients (96.7%). One patient had a small residual lesion on postoperative MRI. Importantly, no postoperative neurological deterioration was observed. Complications were limited to two cases of cerebrospinal fluid (CSF) leakage (6.7%); one resolved after surgical revision and one was managed conservatively. No postoperative infections or hematomas occurred. These findings compare favorably with reported outcomes of open surgery, where GTR rates range from 84% to 93% and complication rates include CSF leaks in 2.7–8%, infections up to 10.7%, and hematomas up to 1.9%.

**Conclusion:**
MISS is a safe and effective approach for intra- and extradural extramedullary spinal tumors, enabling high resection rates with low complication risk. By minimizing tissue trauma and preserving spinal integrity, MISS may provide equivalent oncological control while reducing morbidity compared to traditional open surgery.

DE GRUYTER BRILL

**Rare extraspinal myxopapillary ependymoma in the sacrococcygeal region near the rima ani.**
ID: E-DCK-233

**Wirbelsäulenchirurgie / Spinal Surgery**
**Tumorerkrankungen der Wirbelsäule / Spinal Tumours**

Kushtrim Shala[1], George Campanu[1], Samer Zawy Alsofy[2], Christian Ewelt[2]

[1]St. Elisabeth Hospital Beckum, Klinik für Allgemein- und Viszeralchirurgie, Beckum, Deutschland, [2]St. Barbara-Klinik Hamm GmbH, Klinik für Neuochirurgie, Hamm, Deutschland

**Background:**
Ependymomas are glial tumors usually arising within the central nervous system, but their occurence extradural in the sacrococcygeum is exceptionally rare. Because of their rarity, scral ependymomas are often misdiagnosed and only recognized  after advanced imaging or histopathological confirmation.

**Material and methods:**
**Case Presentation**
Ependymomas are glial tumors usually arising within the central nervous system, but their occurence extradural in the sacrococcygeum is exceptionally rare. Because of their rarity, scral ependymomas are often misdiagnosed and only recognized  after advanced imaging or histopathological confirmation.

**Results:**
A review oft he available literature reveals that only a limited number of primary sacral ependymomas have been reported. Surgical resection remains the treatment of choice, with the extent of excision strongly correlating with long-term outcomes. Adjuvant therapies are reserved for recurrent or incompletely resected tumors.

**Conclusion:**
Primary extradural sacrococcygeal ependymomas are very rare entities, particularly in young patients. Early diagnosis and complete surgical removal are critical for favourable prognosis. Reporting such cases contributes valuable information to the limited literature and helps guide optional management strategies.

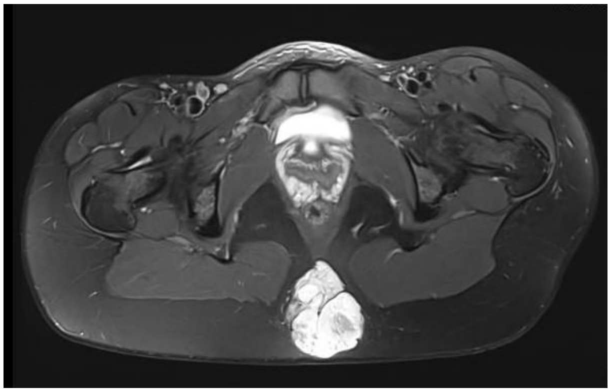

DE GRUYTER BRILL

### Impact of Preoperative Transarterial Embolization in Spinal Metastasis Surgery

ID: E-DCK-448

**Wirbelsäulenchirurgie / Spinal Surgery**
**Tumorerkrankungen der Wirbelsäule / Spinal Tumours**

Moritz Lenschow[1], Maximilian Lenz[2], Marc Schlamann[3], Sergej Telentschak[1], Christoph Kabbasch[3], Niklas von Spreckelsen[4], Krishnan Sircar[2], Maximilian Wankner[1], Nikolaus Kernich[2], Peter Knoll[2], Moritz Perrech[1], Roland Goldbrunner[1], Peer Eysel[2], Volker Neuschmelting[1]

[1]Uniklinik Köln, Zentrum für Neurochirurgie, Köln, Deutschland, [2]Uniklinik Köln, Klinik und Poliklinik für Orthopädie, Unfallchirurgie und Plastisch-Ästhetische Chirurgie, Köln, Deutschland, [3]Uniklinik Köln, Institut für Diagnostische und Interventionelle Radiologie, Köln, Deutschland, [4]Westküstenklinikum Heide, Klinik für Neurochirurgie und Wirbelsäulenchirurgie, Heide, Deutschland

**Background:**
Percutaneous transarterial embolization (PTE) is widely employed to limit intraoperative bleeding during surgery for spinal extradural metastases. Nevertheless, particularly given the associated procedural risks, its impact on clinical outcomes remains insufficiently investigated. We assessed whether PTE is associated with improvement in a composite clinical endpoint.

**Material and methods:**
We undertook a retrospective, propensity score matched cohort study of 666 consecutive patients operated for spinal metastatic disease at an interdisciplinary spine center between 2012 and 2022. Propensity scores incorporated age, sex, Karnofsky Performance Score, tumor vascularity (hyper- vs non-hypervascular), and corpectomy, with 1:1 nearest-neighbor matching using a caliper of 0.2. Hyper-vascular primaries were defined as renal cell or thyroid carcinoma. Patients were stratified by receipt of PTE and compared on a composite endpoint of no major perioperative complications (Clavien–Dindo ≤ II) together with preserved or improved ambulation at last follow-up. Subgroup analyses examined hyper-vascular primaries and corpectomy cases.

**Results:**
PTE was performed in 97 patients; after matching, 194 individuals were analyzed (97 PTE; 97 controls). Median age was 64 years (IQR 56–73), median Karnofsky Performance Score was 70% (IQR 60–80), 62% were male, and median follow-up was 8 months. Corpectomy was undertaken in 53/194 cases (24.3%), and hyper-vascular primaries accounted for 60/194 cases (30.9%). Favorable outcomes were not significantly different between PTE (86/97; 88.7%) and controls (80/97; 82.5%; p=0.220). In hyper-vascular primaries (n=60), favorable outcomes did not differ between PTE (28/30; 93.3%) and non-PTE (26/30; 86.7%; p=0.389). Among corpectomy cases (n=53), favorable outcomes were similar for PTE (22/27; 81.5%) and non-PTE (23/26; 88.5%; p=0.478).

**Conclusion:**
In a propensity score–matched cohort analysis, PTE did not yield a statistically significant improvement in postoperative favourable clinical outcomes, either across the entire cohort or within the hyper-vascular primary and corpectomy groups. Larger prospective studies are needed to ascertain whether selected high-risk populations derive benefit from PTE.

DE GRUYTER BRILL

**Risk–benefit Evaluation of Adjunct Anterior Stabilization for Unstable Thoracolumbar Metastases**
ID: E-DCK-450

**Wirbelsäulenchirurgie / Spinal Surgery**
**Tumorerkrankungen der Wirbelsäule / Spinal Tumours**

Moritz Lenschow[1], Maximilian Lenz[2], Sergej Telentschak[1], Niklas von Spreckelsen[3], Krishnan Sircar[2], Stavros Oikonomidis[2], Nikolaus Kernich[2], Sebastian G. Walter[2], Moritz Perrech[1], Peer Eysel[2], Roland Goldbrunner[1], Volker Neuschmelting[1]

[1]Uniklinik Köln, Zentrum für Neurochirurgie, Köln, Deutschland, [2]Uniklinik Köln, Klinik und Poliklinik für Orthopädie, Unfallchirurgie und Plastisch-Ästhetische Chirurgie, Köln, Deutschland, [3]Westküstenklinikum Heide, Klinik für Neurochirurgie und Wirbelsäulenchirurgie, Heide, Deutschland

**Background:**
Spinal metastases are a frequent sequelae of malignant diseases and often necessitate operative management for progressive vertebral body destruction culminating in mechanical instability. Stability may be restored with dorsal-only constructs or combined dorsoventral fixation. In a predominantly palliative context, the biomechanical advantage of dorsoventral constructs must be weighed against the lesser invasiveness of dorsal-only approaches.

**Material and methods:**
We conducted a retrospective review of 683 patients who underwent surgery for spinal metastatic disease at our center between 2012 and 2022. Eligibility required vertebral body collapse with impending or manifest instability according to the Spinal Instability Neoplastic Score (SINS). Patients were dichotomized to dorsal instrumentation versus combined dorsoventral instrumentation and compared with respect to hospital length of stay and perioperative as well as implant-related complications.

**Results:**
Two hundred eighteen patients met the inclusion criteria. Median age was 63.5 years and median Karnofsky Performance Scale (KPS) was 70%. Impending instability (SINS 7-12) was observed in 45.9%, and high grade Epidural Cord Compression Scale (ESCC) scores 2-3 in 39.9%. Dorsoventral instrumentation was performed in 78 cases (35.8%), whereas 140 cases (64.2%) received dorsal fixation alone. The groups were comparable for age, sex, KPS, ESCC score, overall SINS, and SINS components; for example, de novo deformity occurred in 37.9% of dorsal-only and 42.1% of dorsoventral cases. Median hospital stay was significantly longer after dorsoventral surgery (13 days) than after dorsal procedures (7 days; p=0.031).
Cardiovascular complications were significantly more frequent following dorsoventral (21.8%) than dorsal procedures (9.3%; p=0.010). Implant-related complications were likewise significantly more frequent with dorsoventral (10.3%) than dorsal instrumentation (3.6%; p=0.046). Conversely, revision surgery for complications occurred significantly more often after dorsoventral (16.7%) than dorsal fixation (2.9%; p<0.001). No unplanned secondary corpectomy was required after any case of stand-alone dorsal instrumentation. Rates of thromboembolic events (p=0.102), wound-related complications (p=0.566), and non–wound-related infections (p=0.254) did not differ significantly between the two groups.

Abstracts – DCK 2026 – 143rd Congress of the German Society of Surgery – Leipzig, April 22–24 • DOI 10.1515/iss-2026-2009
Innov Surg Sci 2026; 11 (Special Suppl 1): s428–s437

**Conclusion:**
Operative management of spinal metastases carries substantial morbidity in a population with limited life expectancy and quality of life. In this cohort, complication rates were higher with dorsoventral approaches, and no additional corpectomies were necessary after dorsal instrumentation for metastatic vertebral body destruction.
Overall, these findings suggest that even in the setting of marked vertebral body destruction, dorsal instrumentation may be non-inferior to dorsoventral constructs for achieving spinal stability while incurring a lower complication burden in this cohort with limited overall survival.
